# The Prevalence of Fragility Fractures in a Population of a Region of Southern Italy Affected by Thyroid Disorders

**DOI:** 10.1155/2016/6017165

**Published:** 2016-10-11

**Authors:** Giuseppe Maccagnano, Angela Notarnicola, Vito Pesce, Simona Mudoni, Silvio Tafuri, Biagio Moretti

**Affiliations:** ^1^Orthopaedics Unit, Department of Basic Medical Science, Neuroscience and Sensory Organs, Faculty of Medicine and Surgery, University of Bari, General Hospital, Bari, Italy; ^2^Department of Biomedical Sciences and Human Oncology, Faculty of Medicine and Surgery, University of Bari, General Hospital, Bari, Italy

## Abstract

In the literature there is no clear evidence of a relationship between thyropathies and fragility fractures. The aim of our study is to define the prevalence of thyroid disease in a study sample made up of subjects with fragility fractures and from the same geographical area. We retrospectively studied the “hospital discharge records” (HDR) in the Apulian Database for the period 2008–2013 in order to identify all those patients with fragility fractures that required hospitalization. After detecting the prevalent population, we identified the patients affected by thyroid disease. We observed that, between 2008 and 2013 in Apulia, 16,636 patients were affected by hyperthyroidism. In the same period there were 92,341 subjects with hypothyroidism. The incidence of fragility fractures was 4.5% in the population with hyperthyroidism. As regards the population with hypothyroidism, the incidence of fragility fractures was 3.7%. Furthermore, we assessed the statistical connection between thyroid disease and fragility fractures revealing a higher incidence in patients with hyperthyroidism and clinical hypothyroidism.

## 1. Introduction

Osteoporosis is currently the most common metabolic disease of the skeleton in the world, with an estimated prevalence of approximately 200 million people. The prevalence of the disease increases with an aging population and it is estimated that 30% of European women in menopause are suffering from osteoporosis [1]. The complications of osteoporosis are a major cause of disability and constitute one of the largest items in the budget of health expenditure in several countries, surpassing, according to some authors, myocardial infarction [2]. Fragility fractures (femoral, vertebral, humerus, wrist, tarsus, and metatarsus) are lesions due to mechanical stress not ordinarily resulting in fracture. Moreover, these lesions are caused by low-energy trauma according to National Institute for Health and Care Excellence. Fragility fractures are the most frequent complications of osteoporosis. However, numerous studies have shown that, in patients with low bone mass, all types of fractures are quite frequent. Furthermore, regardless of the skeletal segment concerned, the fracture event increases by 50–100% the likelihood of a subsequent fracture in a different location [3].

In the elderly population, the incidence of fragility fractures and chronic diseases is increasing [4]. In literature there are various works in which the authors have defined the association between osteoporosis and chronic diseases such as diabetes, hypertension, heart disease, and thyroid disease. As for the latter, in many works the association between hyperthyroidism and osteoporosis has been highlighted [5–7], while studies on the relationship between osteoporosis and hypothyroidism [8–10] are controversial and limited. In particular, some authors report an increased incidence of fragility fractures in hypothyroid patients on hormone replacement therapy (levothyroxine) [10, 11]. Thyroid diseases are responsible for metabolic changes that interfere with cardiovascular, psychic, intestinal, muscle, and bone functions [12].

As regards the functional aspect of thyroid diseases, we recognize hypo- and hyperfunctioning. The relationship between thyroid function and phosphocalcium metabolism was reported for the first time in 1891 by Von Recklinghausen, who described hyperthyroidism in osteoporosis [13].

The patient affected with hyperthyroidism characterized by low serum levels of the hormone TSH (≤0.1 IU/L) has an increased risk of vertebral and nonvertebral fractures, respectively, 4.5 and 3.2 times more than those unaffected [14]. The pathogenesis is due to hypofunctioning of pituitary gland. The patients with hyperthyroidism suffer from a wide range of symptoms, for example, anxiety, palpitation, ophthalmopathy, sweating, and slimming while in the patients with hypothyroidism we may describe bradycardia, increased perception of cold, impairment of memory, goiter, lymphoedema of arms, and obesity. In the first case, there emerged hyperproduction of thyroid hormone due to different causes. In the case of hypothyroidism there is a lack of thyroid hormone synthesis with an increase of TSH hormone. The prevalence of hypothyroidism varies between about 0.1 and 2% [15]. The effect of thyroid hormone therapy on BMD shows conflicting results [16–18].

Several authors have underlined the role of replacement therapy as being responsible for the reduction of serum TSH levels which in turn adversely affects bone mineral density. As explained above, it is clear how important it is to define the epidemiological data of the national and regional territory in order to understand in terms of primary prevention and secondary and health economics the problem of fragility fractures in the thyreopatic population. The aim of our study is to determine the prevalence of thyroid disease in the study sample represented by individuals with fragility fractures and from the same geographical area.

## 2. Materials and Methods

We requested the consent of the local ethics committee, and we designed a retrospective observational clinical study. The authors conducted an epidemiological survey on patients who were admitted in different hospitals of the Orthopedics and Traumatology Unit of the Apulia Region. Apulia is a region of Southern Italy with a number of 4,090,266 inhabitants (given Istat updated to 31 December 2013). In order to identify all patients with fragility fractures that required hospitalization, we evaluated the hospital discharge record (HDR) in the period 2008–2013.

In particular, we selected all the Apulia inpatient males over 65 years of age and women over the age of 50 years whose HDR reported domestic accidents (low-trauma energy) in the “trauma field” and who did not die in hospital.

The database of patients with fragility fractures was analyzed using a key linkage individual sanitary code and eliminating all duplicates. This database represented the prevalent population used for the study. The definition of proportion of subjects with fragility fracture and thyroid disease was the primary outcome of the study.

**Figure 1:**
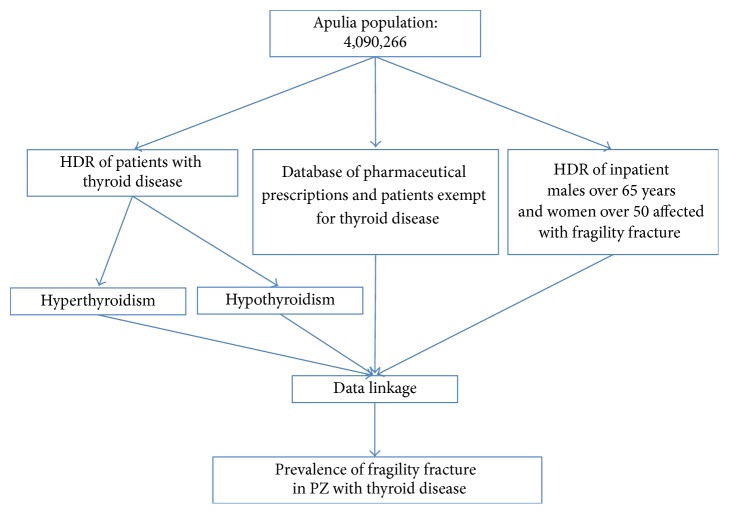
A flow-chart to describe the selection of patients in the period 2008–2013.

The data sources were HDR: database of pharmaceutical prescriptions of medications band A and the database of the Regional Health Service of Apulia regarding patients exempt from paying medications for thyroid disease (Figure 1). A mining algorithm to select the subjects prevailing cohorts suffering from thyroid disease in 2008 was created, considering every person who, in that year, was hospitalized for thyroid disease or reported a prescription charge exemption for the disease or at least a prescription for a tracer drug.

The extracted records were then linked with the “tax code” field; the processing of the data was performed through the WAS MP11 software.

For the definition of the event, the thyroid diseases were reported according to the ICD-IX codes.

In particular, patients with hyperthyroidism were defined as follows:

(i) Every subject of the prevailing population who was hospitalized for a main cause correlated with hyperthyroidism, as follows. 

*HDR Code of Hyperthyroidism*

 242.0 DIFFUSE TOXIC GOITER
 242.1 NODULAR TOXIC GOITER
 242.2 MULTINODULAR TOXIC GOITER
 242.3 ASPECIFIC NODULAR TOXIC GOITER

(ii) Every subject with at least one of the following prescription drugs (ATC A010) ascertained through data linkage with the regional database of pharmaceutical prescriptions and those with the exemption code: 035 (BASEDOW DISEASE AND OTHER FORMS OF HYPERTHYROIDISM) as follows.

*Drugs Prescribed for Hyperthyroidism*

 H03B ANTI THYROID DRUGS
 H03BA THIOURACIL
 H03BA01 METIL THIOURACIL
 H03BA02 PROPYL THIOURACIL
 H03BA03 BENZYL THIOURACIL
 H03BB IMIDAZOLE CONTAINING SULFUR
 H03BB01 CARBIMAZOLE
 H03BB52 TIAMAZOL
 H03BC PERCHLORATE
 H03BC01 POTASSIUM PERCHLORATE
 H03BX OTHER ANTI THYROID PREPARATIONS
 H03BX01 DIIODOTYROSINE
 H03BX02 DIBROMOTYROSINE
 H03CA IODIOTHERAPY

Similarly, patients with hypothyroidism were defined as follows.

(i) Every subject of the prevailing population who was hospitalized for a main cause correlated with hypothyroidism, as follows. 

*HDR Code of Hypothyroidism*

  243 CONGENITAL HYPOTHYROIDISM
244.1 OTHER HYPOTHIROIDISM TYPE DUE TO THYROID ABLATION
244.2 HYPOTHYROIDISM DUE TO IODIO
244.3 OTHER IATROGEN HYPOTHIROIDISM TYPE
244.8 OTHER ACQUIRED HYPOTHIROIDISM TYPE

(ii) Every subject with at least one of the following prescription drugs (ATC A010) ascertained through data linkage with the regional database of pharmaceutical prescriptions and those with the exemption code: 027 (congenital hypothyroidism, SEVERE ACQUIRED HYPOTHYROIDISM) (TSH values greater than 10 mU/L) as follows.

*Drugs Prescribed for Hypothyroidism*

 
  H03AA01 LEVOTHYROXINE SODIUM
  H03AA02 LIOTHYRONINE SODIUM
  H03AA03 COMBINATION OF LEVOTHYROXINE AND LIOTHYRONINE
  H03AA04 TIRATRICOL
  H03AA05 THYROID GLAND PREPARATIONS

Furthermore, the authors defined the subject affected by hypothyroidism who was detected simultaneously in both the HDR database codes and the exemption codes but not in the drug prescription database; the subjects present in database 3 were defined as patients with hypothyroidism in treatment. The databases were linked using as a key linkage individual sanitary code and eliminating all duplicates. Data were stratified by age (50–54 years, 55–59 years, 60–64 years, 65–69 years, 70–74 years, 75–79 years, 80–84 years, and ≥85 years) and by gender (male and female).

## 3. Results

In Apulia, for the period between 2008 and 2013, we revealed 16,636 patients with hyperthyroidism of which 75% were female. The analysis by year class shows a greater distribution in 60–64 years of age range (Table 1).

In the study period, we revealed 92,341 patients with hypothyroidism in Apulia. 84% were female, while the stratification by age showed a greater distribution in the 55–59 years' class (Table 2). In the population of patients affected by hypothyroidism (92,341), 95.1% (87,842) presented clinical hypothyroidism while 4.9% (4,499) presented subclinical hypothyroidism and so were not in drug treatment.

For the period in question, the analysis of data of fragility fractures in the population with hyperthyroidism allowed us to detect an incidence value of 4.5% (741). The most frequent year class was 80–84 years and gender analysis showed a higher incidence in females than in males (85% versus 15%) (Table 3).

As regards the population affected by hypothyroidism, the incidence of fragility fractures was 3.7% (3,450); the most frequent age group was 75–79 years. According to gender analysis, we found a higher incidence in females than in males (90% versus 10%) (Table 4). The incidence of pathologic fractures in patients with subclinical hypothyroidism was 2.6% (118), and in subjects with clinical hypothyroidism it was 3.8% (3,332). The most frequent year class for the former was 60–64 years and for the latter 75–79 years (Table 5). Analyzing the data of fractures regarding the site, the order of frequency did not change between the two groups (hyper- and hypothyroid) (Table 6).

## 4. Discussion

The aim of our study was to determine through observation of a numerically significant sample the real influence of thyroid disease in the Apulian population affected by fragility fractures.

The relationship between hyperthyroidism and bone tissue was defined for the first time in 1890 by the author Von Recklinghausen who described the clinical history of a patient with hyperthyroidism and multiple fractures [19]. By observation of the Apulian population with hyperthyroidism, we defined a gender difference in favour of females and a higher incidence of this disease in the year class 60–64 (Table 1). In accordance with literature, we verified an increase of the incidence value in the first age classes with the maximum value in the range 60–64 [20].

The etiological relationship has been recently confirmed by several authors, who found that the secondary hyperthyroidism in Graves' disease, toxic multinodular goiter, and adenoma of Plummer may modify mineral density and increase risk fracture [21, 22].

Other studies have reported that the effects of hyperthyroidism on metabolism and bone mass are due to an imbalance between osteoblast and osteoclast activity and negative calcium intake. Imbalance causes a reduction of the remodeling process and decreases osteogenesis [23]; negative calcium intake is responsible for decreasing PTH and so modifying the hydroxylation of vitamin D [24].

As for the sample of subjects affected by hypothyroidism, in the period of observation, we found a higher number of subjects than those with hyperthyroidism (92,341 versus 16,636). Also in this case, the females were the most represented (84%), and regarding age, the highest number of cases was in the 55–59 years of age class and so younger than patients with hyperthyroidism, in accordance with the literature [25] (Table 2).

In literature the relationship between hypothyroidism and fragility fracture is not well defined. The role of TSH is controversial; in fact different authors report contradictory effects on bone tissue. In the congenital hypothyroidism, there emerged a decrease in bone development and thyroid hormone administration increased bone ingrowth and mineral density [26, 27]. In other studies, the effects of hormone replacement therapy are contradictory [16, 28].

It is important to distinguish subclinical hypothyroidism and clinical hypothyroidism since in the latter, the subject is under hormone replacement treatment.

Furthermore, we assessed the relationship between thyroid disease and fragility fractures. In the subjects with hyperthyroidism, the incidence of fractures was 4.5%. The gender most represented was the female (Table 3). According to the data of literature we confirm the increased risk of fragility fractures in patients with hyperthyroidism [5]. Moreover in our study we confirm a gender difference in favour of females (Table 3).

As for the sample of the subjects with hypothyroidism, we verified a higher incidence of fractures in the subjects with clinical hypothyroidism with respect to subjects with subclinical hypothyroidism. This value was 3.8% versus 2.6%, respectively. In consideration of the limited difference between the two values, one might believe that this difference is not important; instead by careful analysis there emerged a high difference, in terms of sample number, between two groups. The group of subjects with clinical hypothyroidism is greater than the group with subclinical hypothyroidism (3,332 versus 118) (Table 5).

In the literature, it is reported that, in the presence of high values of TSH (condition of hypothyroidism) or in cases of suppressed TSH (condition of hypothyroidism in treatment), there is an increased risk of fragility fractures [29]. From the data of our study, in consideration of the differences, in terms of increased incidence of fragility fractures in patients with clinical hypothyroidism compared to subclinical, we highlight the importance of the role of hormone replacement therapy. In fact, we may correlate the action of substitution treatment to the increased incidence of fractures and reduced importance of TSH. From our study, there emerged a direct relationship between hyperthyroidism and fragility fractures, confirming the different works in the bibliography; also for the sample of hypothyroidism, there emerged a direct correlation between hypothyroidism and fragility fractures. The authors believe the latter may be due to the lack of screening of osteoporosis in subjects with hypothyroidism. Indeed, while on the one hand, the increased risk of osteoporosis and fragility fractures in the patients with hyperthyroidism is known and for this reason the patients are screened, on the other hand the data in the literature are not yet clear and well defined for subjects with hypothyroidism. As emerged from the observation of our results, the subjects with hypothyroidism are nevertheless affected by fragility fractures. For this reason they should be considered affected by osteoporosis but not under treatment.

The observation of the fracture sites, in both samples, did not show any difference. Moreover the authors found the same order of fracture sites and that the femur represents the main site of fragility fractures in terms of statistical results for both groups.

## 5. Conclusion

Our study is the first that defines the prevalence of fragility fractures in relation to thyroid disease in a population of the same geographical area. The authors, by reaching the main objective and the observation of the Apulian population, confirm the role of thyroid disease as a risk factor in the onset of fragility fractures.

The data confirmed the increased risk of fragility fractures in the population affected by hyperthyroidism and hypothyroidism under treatment. The data also confirm that, besides TSH, hormone replacement therapy also plays an important role in inducing the alterations in bone mineral density. Furthermore, we define an increased risk of fragility fractures in hypothyroidism under treatment. The latter data were interpreted considering the hypothyroid patients with fragility fractures as subjects never screened for osteoporosis and thus affected by osteoporosis but never treated. In consideration of this, it is important to consider also the subjects with hypothyroidism have a risk for the onset of osteoporosis and therefore should be introduced to the diagnostic iter for osteoporotic disease.

## Figures and Tables

**Table 1:** Prevalence of hyperthyroidism, by sex and age group. Apulia, years 2008–2013.

Year class	M	F	Total
50–54	426	1.816	2.242
55–59	584	1.863	2.447
60–64	658	1.799	2.457
65–69	664	1.550	2.214
70–74	611	1.452	2.063
75–79	622	1.599	2.221
80–84	440	1.317	1.757
≥85	212	1.023	1.235

Total %	4.217 (25%)	12.419 (75%)	16.636 100 (%)

**Table 2:** Prevalence of hypothyroidism, by sex and age group. Apulia, years 2008–2013.

Year class	M	F	Total
50–54	2051	14.078	16.129
55–59	2360	14.833	17.193
60–64	2546	14.186	16.732
65–69	2317	11.437	13.754
70–74	1976	9.384	11.360
75–79	1559	6.996	8.555
80–84	1076	4.262	5.338
≥85	666	2.614	3.280

Total %	14.551 (16%)	77.790 (84%)	92.341 (100%)

**Table 3:** Incidence of fragility fractures in patients with hyperthyroidism, by sex and year class. Apulia, years 2008–2013.

Year class	M	F	Total
50–54	3	24	27
55–59	9	37	46
60–64	16	49	65
65–69	10	68	78
70–74	14	68	82
75–79	18	138	156
80–84	25	134	159
≥85	14	114	128

Total	109	632	741

**Table 4:** Incidence of fragility fractures in patients with hypothyroidism, by sex and year class. Apulia, years 2008–2013.

Year class	M	F	Total
50–54	12	231	243
55–59	40	277	317
60–64	40	371	411
65–69	38	404	442
70–74	42	504	546
75–79	60	553	613
80–84	59	461	520
≥85	51	307	358

Total	342	3.108	3.450

**Table 5:** Incidence of fragility fractures in patients with hypothyroidism, subclinical and clinical, by year class. Apulia, years 2008–2013.

Year class	Subjects with subclinical hypothyroidism	Subjects with clinical hypothyroidism	Total
50–54	8	235	243
55–59	17	300	317
60–64	29	382	411
65–69	20	422	442
70–74	11	535	546
75–79	20	593	613
80–84	8	512	520
≥85	5	353	358

Total	118	3332	3450

**Table 6:** Percentage of fragility fractures in patients with hypothyroidism and hyperthyroidism according to the fracture site. Apulia, years 2008–2013.

Fracture side	Patients with hypothyroidism	Patients with hyperthyroidism
Femur	42.9%	55.9%
Humerus	15.2%	13.6%
Forearm	12.8%	10.1%
Lumbar spine	11.2%	9.5%
Tibial pilon	10.4%	5.3%
Dorsal spine	5.6%	4.9%
Tarsus and metatarsus	1.5%	0.5%
Sacrum spine	0.4%	0.2%
